# Community Sampling and Integrative Taxonomy Reveal New Species and Host Specificity in the Army Ant-Associated Beetle Genus *Tetradonia* (Coleoptera, Staphylinidae, Aleocharinae)

**DOI:** 10.1371/journal.pone.0165056

**Published:** 2016-11-09

**Authors:** Christoph von Beeren, Munetoshi Maruyama, Daniel J. C. Kronauer

**Affiliations:** 1 Laboratory of Social Evolution and Behavior, The Rockefeller University, New York, NY, 10065, United States of America; 2 Department of Biology, Ecological Networks, Technical University Darmstadt, 64287 Darmstadt, Germany; 3 The Kyushu University Museum, Hakozaki 6-10-1, Fukuoka, 812–8581 Japan; Smithsonian National Museum of Natural History, UNITED STATES

## Abstract

Army ant colonies host a diverse community of arthropod symbionts. Among the best-studied symbiont communities are those of Neotropical army ants of the genus *Eciton*. It is clear, however, that even in these comparatively well studied systems, a large proportion of symbiont biodiversity remains unknown. Even more striking is our lack of knowledge regarding the nature and specificity of these host-symbiont interactions. Here we surveyed the diversity and host specificity of rove beetles of the genus *Tetradonia* Wasmann, 1894 (Staphylinidae: Aleocharinae). Systematic community sampling of 58 colonies of the six local *Eciton* species at La Selva Biological Station, Costa Rica, combined with an integrative taxonomic approach, allowed us to uncover species diversity, host specificity, and co-occurrence patterns of symbionts in unprecedented detail. We used an integrative taxonomic approach combining morphological and genetic analyses, to delineate species boundaries. Mitochondrial DNA barcodes were analyzed for 362 *Tetradonia* specimens, and additional nuclear markers for a subset of 88 specimens. All analyses supported the presence of five *Tetradonia* species, including two species new to science. Host specificity is highly variable across species, ranging from generalists such as *T*. *laticeps*, which parasitizes all six local *Eciton* species, to specialists such as *T*. *lizonae*, which primarily parasitizes a single species, *E*. *hamatum*. Here we provide a dichotomous key along with diagnostic molecular characters for identification of *Tetradonia* species at La Selva Biological Station. By reliably assessing biodiversity and providing tools for species identification, we hope to set the baseline for future studies of the ecological and evolutionary dynamics in these species-rich host-symbiont networks.

## Introduction

"Die Wechselbeziehungen, die zwischen den Ameisen, […], und ihren fremden Gesellschaftern in allen Welttheilen obwalten, sind eines der reichhaltigsten und dankbarsten Forschungsgebiete der Biologie. […] Um dem Wissenschaftlichen Studium jener Wechselbeziehungen eine feste Grundlage zu geben, ist es aber vor Allem nöthig, genau festzustellen, bei welchen Arten von Wirthen die einzelnen Gastarten gesetzmässig vorzukommen pflegen."

Erich Wasmann 1894

("The interactions that occur between ants […] and their foreign associates across the world constitute one of the richest and most rewarding research areas in biology. […] However, to provide a firm foundation for the scientific study of these interactions, it is of primary importance to precisely determine with which host species the different guest species tend to be associated.")

Erich Wasmann, a Jesuit priest and Austrian entomologist, was dedicated to the study of ‘ant guests’. He published the first comprehensive catalogue of more than 1,000 ant-associated species, the vast majority of them arthropods [1]. Since his pioneering work, the known diversity of ant-associated arthropods has been extended further [2,3]. These guests, when regularly associated with, rather than merely accidentally present with ants, are known as myrmecophiles [4]. Estimates of myrmecophile diversity range from 10,000 to 100,000 arthropod species in over 100 families [5–7]. Given their diversity, the myrmecophiles in and around ant colonies provide a good opportunity to study the structure and determinants of complex host-symbiont communities [8]. Army ants and their associated guests represent particularly species-rich host-symbiont assemblages [3,9,10]. For example, several hundred myrmecophile species are associated with a single army ant species, the infamous swarm-raider *Eciton burchellii* [11]. It is clear, however, that a large proportion of guest diversity is still unknown, and that the taxonomy for many groups remains unsettled [11,12]. Furthermore, host preferences and co-occurrence patterns are poorly understood, because rather than sampling myrmecophiles systematically, guests have been opportunistically collected across many different locations [2,8,11]. The first step toward understanding the ecological and evolutionary interactions between army ants and their myrmecophiles is therefore an accurate assessment of species diversity, combined with systematic community sampling within a given population.

**Fig 1:**
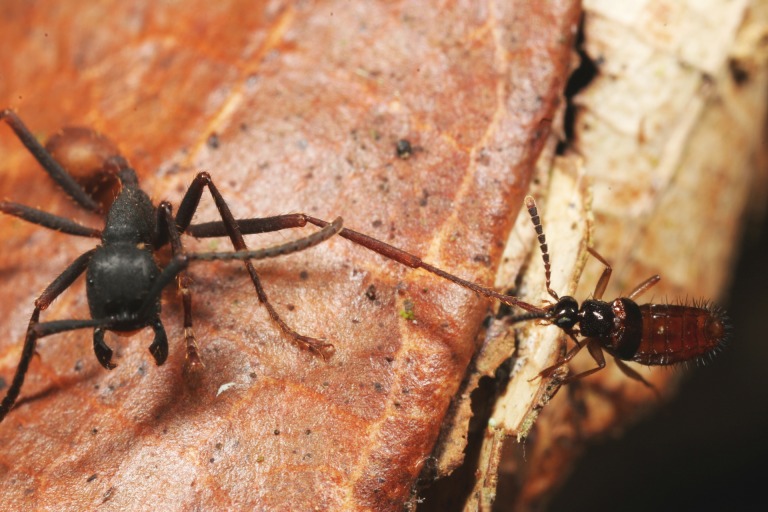
*Tetradonia* beetles are predators of Neotropical army ants. Shown is a *Tetradonia* beetle attacking an adult *Eciton burchellii* worker during a colony emigration. *Tetradonia* beetles are the only army ant myrmecophiles known to regularly kill and feed on adult workers. Photo: Daniel Kronauer; Parque Nacional Henri Pittier, Venezuela.

Inspired by Carl Rettenmeyer’s seminal work on the guests of Neotropical army ants [9,11,13–16], we started systematically surveying the arthropod symbiont community of the six local *Eciton* army ant species at La Selva Biological Station (LSBS) in Costa Rica. We have already reported cryptic species diversity in rove beetles of the genus *Vatesus*, demonstrating the need for correct assessment of species boundaries in order to reliably determine host specificity [12], one of the most fundamental symbiont life history traits [17,18]. Here we applied the same community-based sampling, combined with an integrative taxonomic approach, to describe the species diversity and host specificity of the rove beetle genus *Tetradonia* Wasmann, 1894 at LSBS. Members of this genus are confined to the New World, where they are associated with various army ant genera of the tribe Ecitonini (Formicidae, Dorylinae) [19]. The genus currently contains 35 described species, nine of which are recorded from Costa Rica [19–21]. *Tetradonia* beetles are the only described arthropod symbionts that regularly prey on adult army ant workers, mostly on dying or injured ones [11] (Fig 1). Host records indicate that interactions range from species that are associated with a single host species to host generalists [19]. For example, *Tetradonia marginalis*, a common guest of *Eciton* ants, seems to be associated with seven different army ant species [19]. However, the available host records are a typical example of the scattered collection efforts for army ant symbionts, and species identifications are often questionable. Consequently, the true level of host specificity remains uncertain.

During our survey of *Eciton* army ants and their arthropod guests at LSBS, we collected several thousand specimens of *Tetradonia* beetles, of which we analyzed 362 individuals for the current study. A combination of morphological and genetic data revealed five distinct species. Three of them were previously recognized species, whereas two were hitherto undescribed. We provide species descriptions for the five species and a dichotomous key to the *Eciton*-associated *Tetradonia* species at LSBS. Host specificity was variable and ranged from host generalists to host specialists. Our study highlights the need for standardized sampling in combination with accurate assessment of species boundaries to correctly describe the existing interactions in species-rich host-symbiont communities.

## Methods

### Collection and depository of specimens

We conducted a community survey of *Eciton*-associated myrmecophiles in the tropical rainforest at LSBS, Costa Rica (N1025.847 W84 00.404, altitude 67 m asl), from February to April 2013 and March to April 2014 (15 weeks total). We systematically collected myrmecophiles from all six local *Eciton* species during colony emigrations: *E*. *burchellii foreli* Mayr, 1886 [22], *E*. *hamatum* Fabricius, 1781 [23], *E*. *vagans angustatum* Roger, 1863 [24], *E*. *dulcium crassinode* Borgmeier, 1955 [25], *E*. *mexicanum* s. str. Roger, 1863 [24], and *E*. *lucanoides conquistador* Weber, 1949 [26] (Table 1; for more details see [12]). We also collected myrmecophiles from one *Neivamyrmex gibbatus* Borgmeier, 1953 [27] and one *Neivamyrmex pilosus mexicanus* Smith, 1859 [28] colony emigration (Table 1). In addition, we opportunistically collected myrmecophiles from *Eciton* refuse sites and raids (Table 1; for detailed records see S1 Table). Army ant species were identified using the identification keys of Watkins 1982 [29] and Longino 2007 [30]. Subspecies assignments are additionally based on the distribution maps provided by Watkins 1976 [31]. Voucher army ant workers of all species are stored in CvB’s personal collection. *Tetradonia* specimens are stored at the Kyushu University Museum, Fukuoka, Japan (KUM), the Connecticut State Museum of Natural History, CT, USA (CSMNH), the Field Museum of Natural History, Chicago, IL, USA (FMNH), and the private collection of CvB (CvB) (for details see S1 Table). In addition, 111 voucher images from 22 *Tetradonia* specimens are available in the Barcoding of Life database (S1 Table). Research and specimen export permits for Costa Rica were issued by the 'Ministry of the Environment, Energy and Technology' (MINAET; permit numbers: 192-2012-SINAC and R-009-2014-OT-CONAGEBIO).

**Table 1:** Overview of samples. Shown is the number of host emigrations, raids and refuse deposits from which *Tetradonia* beetles were collected and analyzed. Numbers in parentheses give the number of different colonies of a given host species. Note that the focus of the study was to systematically sample host emigrations, while specimens were only collected opportunistically from raids and refuse deposits. Detailed sample information is given in S1 Table.

	Myrmecophiles collected from…				
Host species	emigrations	raids	refuse deposits	Total no. of colonies	No. of colonies with *Tetradonia*
*Eciton burchellii foreli*	15 (12)	7 (6)	2 (2)	13	13
*Eciton dulcium crassinode*	12 (11)	0	1 (1)	11	10
*Eciton hamatum*	12 (10)	4 (3)	2 (2)	13	13
*Eciton lucanoides conquistador*	2 (2)	2 (1)	0	3	3
*Eciton mexicanum* s. str.	14 (11)	1 (1)	0	11	7
*Eciton vagans angustatum*	9 (8)	0	0	8	6
*Neivamyrmex gibbatus*	1 (1)*	0	0	1	1
*Neivamyrmex pilosus mexicanus*	1 (1)*	0	0	1	1

* myrmecophiles were only collected for about 1h rather than from entire emigrations

### Molecular protocol

The high diversity of *Eciton*-associated myrmecophiles required an approach to efficiently sort specimens into distinct groups (candidate species) for subsequent study by alpha taxonomists. We thus applied a molecular pre-screening method to assess diversity and detect possible species boundaries in *Tetradonia*. We follow Ernst Mayr’s classical ˈBiological Species Conceptˈ, i.e. a lack of gene flow between sympatric populations defines separate species [32]. We therefore consider distinct genetic clades in sympatric populations as separate species.

DNA from whole specimens was extracted with the QIAGEN DNeasy Tissue Kit for 96-well-plates following the standard protocol except for a shortened digestion step of 2–3 h. All specimens were preserved during this process and are kept as vouchers (for depository information see S1 Table). The mitochondrial *cytochrome oxidase I* (*COI*) barcode region [658 base pairs (bp)] was amplified in standard polymerase chain reactions (PCRs). We attempted to cover the entire morphological diversity of *Tetradonia* specimens for barcoding by initial visual inspection of all specimens encountered in a given host colony. On average, seven *Tetradonia* specimens per *Eciton* colony were analyzed using DNA barcodes (N = 362 *Tetradonia* specimens in total; for numbers of specimens analyzed per colony see S1 Table).

It can be problematic to use mitochondrial DNA alone for a genetic assessment of species boundaries (e.g., [33]), and we therefore supplemented the *COI* data with portions of the nuclear genes *wingless* (*wg*; 469 bp) and *CAD* (476 bp) for a subset of specimens. Because all specimens were collected at a single location, we considered distinct mitochondrial clusters that also showed a clear separation at nuclear loci, i.e. that showed a lack of nuclear gene flow and therefore lack of sexual recombination, as distinct species (see also [33]). For the collection of nuclear sequence data we chose 8–24 specimens of each mitochondrial cluster. These specimens represented all of the different *COI* haplotypes. PCRs were setup as described previously [12]. PCR product purification and sequencing were outsourced to the companies Macrogen USA and Eton Bioscience. All PCR products were sequenced in both directions. In cases of low quality reads, PCR and sequencing were repeated. PCR primers and annealing temperatures are given in S2 Table.

### Morphological protocol

The terminology and procedures used in this study follow those described previously [34,35]. Line drawings were produced using a Nikon 50i microscope equipped with a Nikon Y-IDT drawing tube. Permanent mounts were made for genital parts, which were first soaked in 10% potassium chloride solution, washed in water and then slowly dehydrated in ethanol (for details see [12]). Dehydrated specimens were then embedded in Euparal mounting medium (Chroma-Gesellschaft), and when appropriate, further dissections of genital parts were made within Euparal on a slide or halved glass cover slip glued onto a halved paper glue board (for details see [36]).

### Nomenclatural acts

The electronic edition of this article conforms to the requirements of the amended International Code of Zoological Nomenclature (ICZN), and hence the new names contained herein are available under that Code from the electronic edition of this article. This published work and the nomenclatural acts it contains have been registered in ZooBank, the online registration system for the ICZN. The ZooBank Life Science Identifiers (LSID) can be resolved and the associated information viewed through any standard web browser by appending the LSID to the prefix “http://zoobank.org/”. The LSID for this publication is: urn:lsid:zoobank.org:pub:C83ED521-37C4-4DE3-81DE-67F061CBC8BC. The electronic edition of this work was published in a journal with an ISSN, and has been archived and is available from the following digital repositories: PubMed Central, LOCKSS.

### Data analyses

Collection data, DNA extraction, PCR settings, and sequencing results were tracked for individual samples using the software Geneious® R9 (version 9.1.5) with the plugin ‘biocode’ (version 3.0.0) [37]. Geneious® was also used to trim sequences and to calculate Neighbor-Joining (NJ) trees with bootstrap support (1,000 replicates) based on Tamura-Nei distances. NJ trees were used to screen for distinct genetic clusters within the dataset. Branches of bootstrap support values ≤ 50 were collapsed and shown as a polytomy. A ‘Randomized Axelerated Maximum Likelihood’ (RAxML) tree based on concatenated *COI*, *wg*, and *CAD* sequences was created using the software Geneious R9 (version 9.1.5) with the plugin RAxML (version 7.2.8). GTR GAMMA was chosen as the best-scoring model as assessed by MEGA6 [38]. For *COI* data, we calculated p-distances in pairwise comparisons (with gaps being deleted), the number of parsimony informative sites and the number of singletons in MEGA 6 [38]. The software ‘barcoding with LOGic’ (BLOG 2.0) was used with default settings to find a logic formula of unique nucleotides in *COI* sequences for each species [39]. Within a given *Tetradonia* species, about 60% of sequences were used as training set and about 40% of sequences as test set. *COI* sequences shorter than the 658bp full length read were omitted from this analysis. All sequences have been uploaded to GenBank, with accession numbers listed in S1 Table.

## Results

### Molecular species identification

We used *COI* barcodes as a first indicator of species diversity in *Tetradonia*. *COI* barcodes were obtained from 362 *Tetradonia* specimens (for details see S1 Table). Most sequences were 658bp full length reads, while nine sequences had to be trimmed at one end due to low quality sequencing signals (range 8-33bp trimmed).

**Fig 2:**
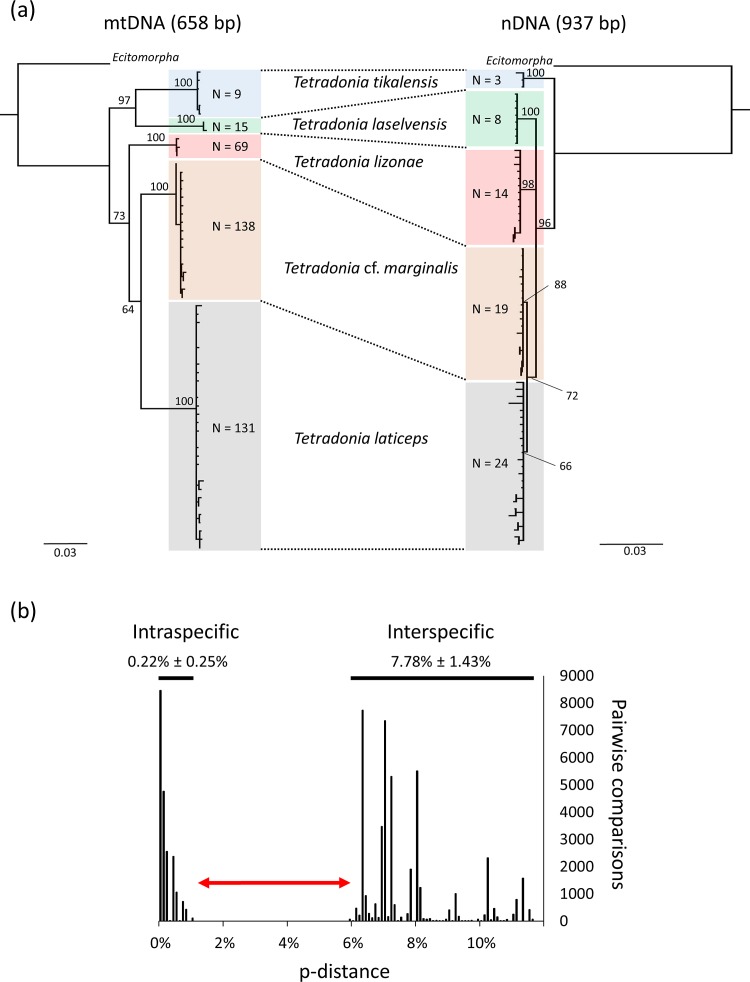
Genetic assessment of species boundaries in a community of *Tetradonia* beetles. (a) NJ trees based on Tamura-Nei distances (scale bars) for both mitochondrial (*COI* sequences, left) and nuclear DNA sequences (concatenated *wg* and *CAD* sequences, right) reveal the same five genetic clusters (i.e. candidate species), which are depicted by separately colored boxes. *Ecitomorpha arachnoides* (Staphylinidae: Aleocharinae: Ecitocharini), collected in an emigration of *Eciton burchellii foreli*, served as outgroup. Numbers in boxes show the numbers of analyzed specimens. Bootstrap support values (1,000 replicates) for major nodes are shown. (b) Histogram showing intra- and inter-specific p-distances between *Tetradonia* mitochondrial *COI* sequences. P-distances give the proportion of bases that differ in pairwise-comparisons. A barcode gap (red double arrow) separates maximum distances within- from minimum distances between species. Mean ± SD p-distances within- and between groups are shown. Abbreviations: bp = base pairs, mtDNA = mitochondrial DNA, nDNA = nuclear DNA.

NJ trees of mitochondrial and nuclear sequences revealed the same five clusters, indicating the existence of five genetically isolated species (Fig 2A). These clusters were also recovered in a RAxML tree of concatenated *COI*, *wg*, and *CAD* sequences (S1 Fig). Genetic clusters were then identified as the following species (see morphological descriptions below): *T*. *lizonae* sp. nov., *T*. *laselvensis* sp. nov., *T*. *tikalensis* Jacobson & Kistner, 1998 [19], *T*. *laticeps* Jacobson & Kistner, 1998 [19], and *T*. cf. *marginalis* Reichensperger, 1935 [40].

The five genetic clusters comprised 63 *COI* haplotypes with no haplotype overlap between clusters (within species: *T*. *lizonae* = 3 haplotypes, N = 69 specimens sequenced; *T*. *laselvensis* = 2 haplotypes, N = 15 specimens sequenced; *T*. *tikalensis* = 6 haplotypes, N = 9 specimens sequenced; *T*. *laticeps* = 31 haplotypes, N = 131 specimens sequenced; *T*. cf. *marginalis* = 21 haplotypes, N = 138 specimens sequenced). Within species, DNA barcodes contained 1, 0, 5, 16, and 5 variable and parsimony-informative sites, and 1, 1, 3, 13, and 15 singletons, respectively. Similarly, there was no overlap in *CAD* and *wg* alleles between *Tetradonia* species (number of *CAD*/*wg* sequences: *T*. *lizonae* = 22/24; *T*. *laselvensis* = 9/9; *T*. *tikalensis* = 3/3; *T*. *laticeps* = 30/28; *T*. cf. *marginalis* = 30/29).

The distribution of intraspecific- and interspecific genetic p-distances in pairwise comparisons showed a clear gap between maximum intraspecific- and minimum interspecific genetic distances of mitochondrial sequences (Fig 2B). Such 'barcoding gaps' further support the presence of distinct species (but see [33]).

A high classification rate in character-based DNA analysis of mitochondrial sequences also supported the genetic disparity of the five previously detected genetic clusters. Following initial training runs, the five *Tetradonia* clusters were defined by the following nucleotide formulas by BLOG: *T*. *lizonae*: T at position 55; *T*. *laselvensis*: G at position 46; *T*. *tikalensis*: C at position 46; *T*. *laticeps*: G at position 322; *T*. cf. *marginalis*: G at position 334. These formulas achieved a 100% correct classification rate for all *Tetradonia* species except for *T*. *laticeps* (98%), where one individual could not be classified.

### Morphological descriptions and host records

Pace [41] stated that the New World genus *Tetradonia* could be a synonym of the Old World genus *Orphnebius* Motschulsky, 1858, and transferred *T*. *marginalis* Reichensperger, 1935 to *Orphnebius*, referring to the similarity in mouthpart structures [41]. We do not agree with this conjecture, as the mouthpart structures of *Tetradonia* are actually quite different from those of *Orphnebius*, especially in the non-elongate galea and lacinia of the maxillae. In particular, molecular phylogenies of Aleocharinae have shown that *Tetradonia* is only distantly related to *Orphnebius*. While the genus *Orphnebius* belongs to the tribe Lomechusini, *Tetradonia* in fact belongs to a separate, unnamed tribe (informally known as "false Lomechusini") nested within the tribe Athetini [42,43]. We thus regard *Tetradonia* as a valid genus, and in the following describe the five species associated with *Eciton* army ants at LSBS.

### *Tetradonia lizonae* von Beeren & Maruyama sp. nov. (LSID: urn:lsid:zoobank.org:act:5B855549-B67F-430E-A613-EB101E77917C)

**Etymology:** Dedicated to Sofia Lizon à l'Allemand, wife of CvB, for her endless support of CvB’s myrmecophile research.

**Type series:** Holotype, male, Costa Rica: Sarapiquí, Puerto Viejo, La Selva Biological Station, 21.III.2013, leg. C. von Beeren and D. Kronauer (cvb451tetr007), *Tetradonia lizonae* C. von Beeren & M. Maruyama des. 2016 (KUM). **Paratypes**. 22 specimens with same collection location as holotype and various collection dates: 08.II.2013: cvb010tetr002, cvb010tetr003, cvb010tetr009, cvb010tetr010, cvb010tetr011 (all KUM); 12.II.2013: cvb038tetr014 (FMNH); 17.III.2014: cvb316tetr003, cvb316tetr009, cvb316tetr010 (all KUM); 19.III.2013: cvb382tetr002, cvb382tetr005 (both KUM); 25.III.2013: cvb433tetr009 (KUM); 29.III.2013: cvb451tetr001 (CSMNH), cvb451tetr002 (CvB), cvb451tetr006 (FMNH), cvb451tetr010 (KUM); 02.IV.2013: cvb488tetr001 (KUM); 07.IV.2013: cvb541tetr010 (KUM); 23.III.2014: cvb603tetr003 (KUM), cvb606tetr002 (KUM), cvb606tetr004 (CSMNH), cvb606tetr007 (KUM). A detailed list with collection and depository information is given in S1 Table.

**Fig 3:**
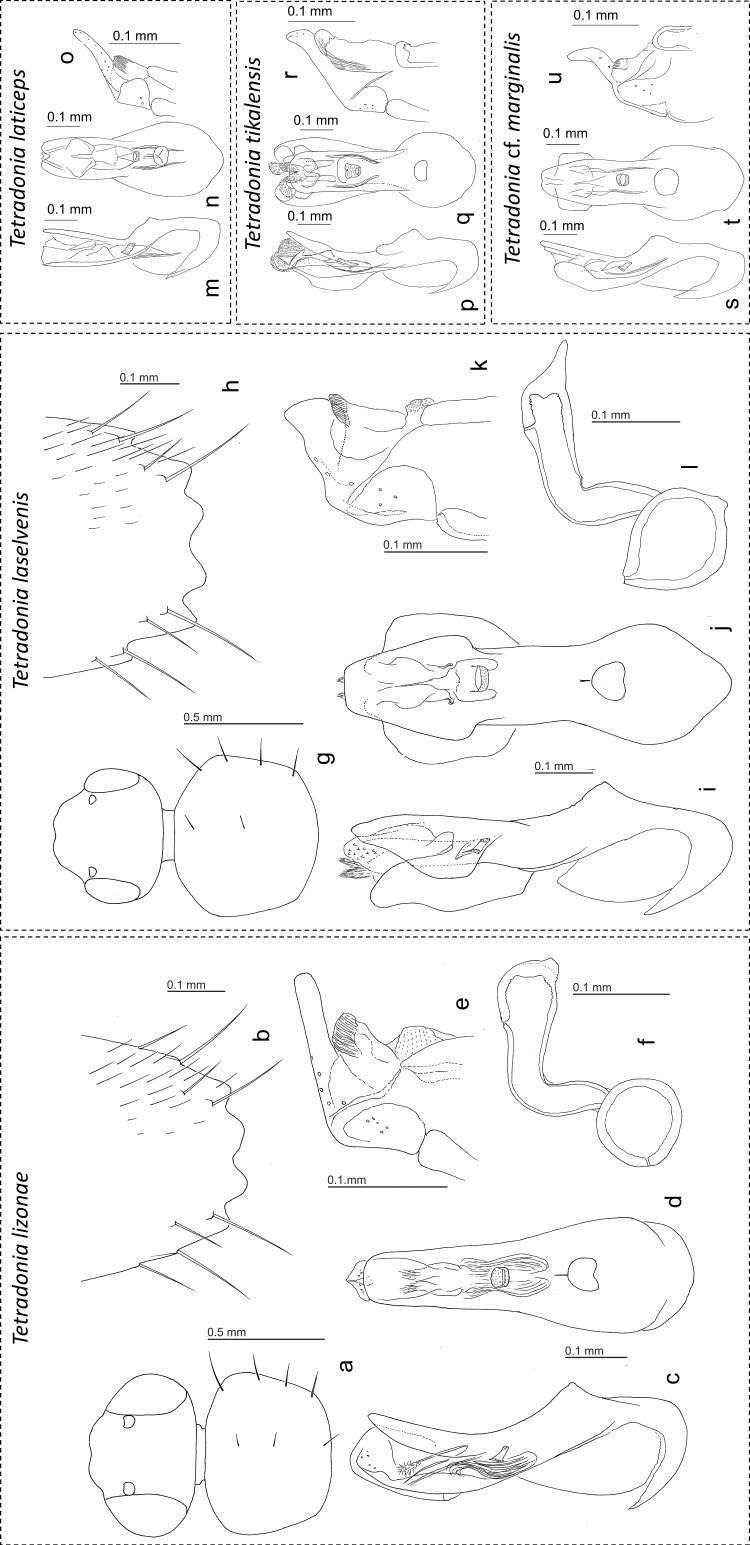
**Drawings of different body parts of *Tetradonia lizonae* (a-f)**, ***T*. *laselvensis* (g-l), *T*. *laticeps* (m-o), *T*. *tikalensis* (p-r) and *T*. cf. *marginalis* (s-u).** Head and pronotum (a, g); male tergite VIII (b, h); median lobe of aedeagus in lateral view (c, i, m, p, s); median lobe of aedeagus in ventral view (d, j, n, q, t); apex of paramere (e, k, o, r, u); spermatheca (f, l).

**Description:** Body small, 3.9–4.4 mm; abdomen and elytra lacking spines. Uniformly reddish brown but head blackish brown. Head capsule transverse (Fig 3A), as wide as pronotum; eyes extremely large, occupying entire sides of head (Fig 3A), 0.8 times as long as head, 0.25 times as wide as head; surface smooth, densely covered with recumbent setae, with several long macrosetae along eyes; antennae long, as long as head, pronotum and elytra combined; all segments longer than wide; segment IX very slightly shorter than X; segment XI shorter than IX and X combined. Pronotum transverse (Fig 3A), widest around anterior 1/5, with 3 small macrosetae along mid line and 4 long macrosetae along lateral margin (Fig 3A); lateral and posterior margins distinctly margined; surface smooth, moderately covered with recumbent setae. Elytra weakly granulate-punctate, shining, moderately covered with recumbent setae, with 1 macroseta near scutellum and 3 macrosetae along lateral margin. Abdomen with tergite VI with 3 macrosetae; tergite VII with 4 macrosetae, without raised area; tergite VIII with 4 macrosetae (Fig 3B), apical emarginations shallow; sternite VIII with 7 macrosetae. Median lobe of aedeagus rather generalized, drop-shaped (Fig 3C and 3D), slightly truncate at apex in parameral view; apical lobe of paramere elongate (Fig 3E). Spermatheca with basal part spherical (Fig 3F); apical part with a small projection at apex.

**Measurements:** Body length, 3.9–4.4 mm; fore body length (from apex of clypeus to apices of elytra), 1.9–2.0 mm; head width, 0.75–0.76 mm; pronotal length, 0.59–0.63 mm; pronotal width, 0.75–0.76 mm.

**Diagnosis:** This species is similar to *T*. *laticeps* in eye size and weakly-punctate elytral surface, but distinguished from it by the tergite VII being without raised area in males; by the narrower pronotum width; by the apical part of the aedeagal median lobe being slightly curved to the paramere; the apical lobe of the paramere being straight; and the basal part of the spermatheca being spherical.

**Distribution:** Only known from La Selva Biological Station.

**Fig 4:**
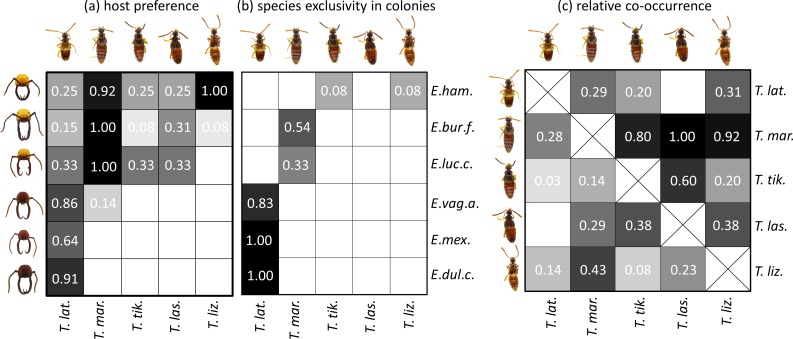
Host preference, colonies exclusively occupied by a single *Tetradonia* species, and relative co-occurrence of *Tetradonia* species at La Selva Biological Station, Costa Rica. (a) White numbers in cells depict host preference (or beetle prevalence) evaluated as the proportion of colonies of a given host species in which a given *Tetradonia* species was collected. (b) Species exclusivity in colonies reflects the proportion of colonies of a given host species in which we found only a single *Tetradonia* species, relative to the total number of sampled colonies of that host, but excluding those colonies in which no *Tetradonia* specimens were found at all (see Table 1). For example, we exclusively found *T*. *laticeps* in 5 colonies out of 8 *E*. *vagans angustatum* colonies sampled, and 6 sampled *E*. *vagans angustatum* colonies contained some *Tetradonia* beetles (i.e., 5/6 = 0.83). (c) To quantify the level of relative co-occurrence of different *Tetradonia* species, we first counted the number of host colonies that were simultaneously parasitized by a given species pair (for absolute number of co-occurrence events see S2 Fig). To account for differences in species prevalence, we divided the total number of co-occurrence events for a given species pair either by the number of host colonies in which the more prevalent partner (lower left) or the less prevalent partner (upper right) was found. For instance, out of the 5 colonies in which *T*. *tikalensis* was found, it co-occurred in 4 of them with *T*. cf. *marginalis*, the latter of which was found in a total of 28 colonies (i.e., standardized for more prevalent partner: 4/28 = 0.14; standardized for less prevalent partner: 4/5 = 0.80). Differential shading corresponds to the white numbers in cells ranging from white (value 0) to black (value 1). Photographs depict frontal head views of *Eciton* soldier workers and dorsal views of *Tetradonia* beetles for the different species. Abbreviations: *E*. *ham*. = *Eciton hamatum*, *E*. *bur*. *f*. = *Eciton burchellii foreli*, *E*. *luc*. *c*. = *Eciton lucanoides conquistador*, *E*. *vag*. *a*. = *Eciton vagans angustatum*, *E*. *mex*. = *Eciton mexicanum* s. str., *E*. *dul*.*c*. = *Eciton dulcium crassinode*, *T*. *lat*. = *Tetradonia laticeps*, *T*. *mar*. = *Tetradonia* cf. *marginalis*, *T*. *tik*. = *Tetradonia tikalensis*, *T*. *las*. = *Tetradonia laselvensis*, *T*. *liz*. = *Tetradonia lizonae*.

**Host records:** The analyzed *T*. *lizonae* specimens were collected in host emigrations (N = 60 specimens) and host raiding columns (N = 9 specimens). Out of 69 analyzed specimens, 68 were associated with *E*. *hamatum* and only one individual was walking in the center of an *E*. *burchellii foreli* emigration (Fig 4). The species showed a high prevalence in *E*. *hamatum* colonies at the study site. Every sampled *E*. *hamatum* colony (N = 12) contained *T*. *lizonae* specimens (Fig 4A). *Tetradonia lizonae* was found together in host colonies with each of the four other *Tetradonia* species, most frequently together with the abundant species *T*. cf. *marginalis* (Fig 4C). Interestingly, *T*. *lizonae* was the only *Tetradonia* species at La Selva that ran within the center of ant emigration columns among host workers. A detailed list of host records can be found in S1 Table.

### *Tetradonia laselvensis* Maruyama & von Beeren sp. nov. (LSID: urn:lsid:zoobank.org:act:54140F81-D58E-4A24-817F-895E4096D6F7)

**Etymology:** Named after the type locality, La Selva Biological Station.

**Type series:** Holotype, male, Costa Rica: Sarapiquí, Puerto Viejo, La Selva Biological Station, 29.III.2013, leg. C. von Beeren and D. Kronauer (cvb541tetr007), *Tetradonia laselvensis* M. Maruyama & C. von Beeren des. 2016 (KUM). **Paratypes**. 13 specimens with same collection location as holotype and various collection dates: 21.II.2013: cvb104tetr001 (CSMNH), cvb104tetr005 (KUM); 03.III.2013: cvb176tetr001, cvb176tetr003 (both KUM); 16.III.2013: cvb308tetr006, cvb308tetr007, cvb308tetr008 (all KUM); 21.III.2013: cvb399tetr004 (CSMNH); 06.IV.2013: cvb529tetr001, cvb529tetr003, cvb529tetr005 (all KUM); 08.IV.2013: cvb572tetr006 (FMNH); 23.III.2014: cvb606tetr003 (FMNH). A detailed list of specimens together with collection and depository information is given in S1 Table.

**Description:** Small, spineless species. Reddish brown but head, pronotum and elytra brown; antennae brown but segment I and XI yellowish brown. Head capsule slightly transverse (Fig 3G), narrower than pronotum; eyes moderate in size, half as long as head (Fig 3G), 0.18 times as wide as head; surface smooth, densely covered with recumbent setae, with several short macrosetae along eyes; antennae moderate in length, shorter than head, pronotum and elytra combined; all segments longer than wide; segment IX shorter than X; segment XI shorter than IX and X combined. Pronotum transverse (Fig 3H), widest around anterior 1/4, with 2 small macrosetae along mid line and 4 long macrosetae along lateral margin; lateral and posterior margins distinctly margined; surface smooth, moderately covered with recumbent setae. Elytra strongly granulate-punctate, shining, moderately covered with recumbent setae, with 1 macroseta near scutellum and 3 macrosetae along lateral margin. Abdominal tergite VI with 3 macrosetae; tergite VII with 4 macrosetae, with a small, transverse raised area postero-medially; tergite VIII with 4 macrosetae (Fig 3H), apical emarginations shallow; sternite VIII with 7 macrosetae. Median lobe of aedeagus with apical part strongly expanded (Fig 3I and 3J), apically truncate in parameral view; apical lobe of paramere (Fig 3K) thickened at apex. Spermatheca with basal part lemon-shaped (Fig 3L); apical part with a long, apically acute projection at apex (Fig 3L).

**Measurements:** Body length, 3.8–4.3 mm; fore body length (from apex of clypeus to apices of elytra), 1.8–2.0 mm; head width, 0.66–0.71 mm; pronotal length, 0.61–0.68 mm; pronotal width, 0.72–0.81 mm.

**Diagnosis:** This species is similar to *T*. *newtoni* Jacobson & Kistner, 1998 [19] in granulate-punctate elytral surface and thickened apex of apical lobe of paramere, but distinguished from it by fewer macrosetae on sternite VIII; the apical part of the aedeagal median lobe being expanded laterally; the apical part of the spermatheca being with a long projection.

**Distribution:** Only known from La Selva Biological Station.

**Host records:** Using our visual and genetic screening, we only found 15 individuals of *T*. *laselvensis*, which is apparently rare compared to *T*. cf. *marginalis*, *T*. *laticeps*, and *T*. *lizonae*. Ten of the analyzed specimens were collected in host emigrations and five in host raids (S1 Table). Given its rarity, our assessment of host preferences should be treated as preliminary. We found it in moderate prevalence with the following three *Eciton* species (Fig 4A): *E*. *burchellii foreli* (9 individuals from 4 out of 13 colonies), *E*. *hamatum* (4 individuals from 3 out of 12 colonies), and *E*. *lucanoides conquistador* (4 individuals from 1 out of 3 colonies). *Tetradonia laselvensis* co-occurred in host colonies with all other *Tetradonia* species except *T*. *laticeps* (Fig 4C). A detailed list of host records can be found in S1 Table.

#### *Tetradonia tikalensis* Jacobson & Kistner, 1998

*Tetradonia tikalensis* Jacobson & Kistner, 1998: 187 (original description) [19]; Hlaváč et al., 2011: 81 (catalogue) [21].

**Specimens examined:** 9 specimens were morphologically examined. They were collected at LSBS on various collection dates: 07.II.2013: cvb004tetr001 (KUM); 08.II.2013: cvb010tetr004 (KUM); 14.II.2013: cvb048tetr006 (KUM); 21.II.2013: cvb104tetr008 (KUM); 21.III.2013: cvb399tetr003 (KUM), cvb399tetr008 (CvB); 08.IV.2013: cvb572tetr005 (CSMNH), cvb572tetr007 (FMNH), cvb572tetr008 (CSMNH). A detailed list of specimens together with collection and depository information is given in S1 Table.

**Diagnosis:** Reddish brown but head, pronotum and apical halves of elytra darker; eyes moderate in size; antennae short, shorter than head, pronotum and elytra combined; segments VII-X as long as wide or slightly longer than wide; elytral surface smooth. Aedeagus and paramere are illustrated in Fig 3.

**Distribution:** Guatemala (original description, type locality), Costa Rica (new record).

**Host records:** Eight of the analyzed *T*. *tikalensis* specimens were collected from host colony emigrations and one individual from a host raid. This species was previously described to be associated with *E*. *burchellii* and *E*. *hamatum* [19]. We confirm these host associations and add a new host record (Fig 4): we found one specimen in an *E*. *lucanoides conquistador* emigration. As we only found 9 individuals in total with our visual and genetic screening, host specificity and preference is difficult to assess (Fig 4; S2 Fig). A detailed list of host records can be found in S1 Table.

#### *Tetradonia laticeps* Jacobson & Kistner, 1998

*Tetradonia laticeps* Jacobson & Kistner, 1998: 192 (original description) [19]; Hlaváč et al., 2011: 80 (catalogue) [21].

**Specimens examined:** 20 specimens were morphologically examined. They were collected at LSBS on various collection dates: 07.II.2013: cvb002tetr003 (KUM); 10.II.2013: cvb020tetr006 (KUM); 12.II.2013: cvb038tetr010 (KUM); 14.II.2013: cvb048tetr007 (KUM); 18.II.2013: cvb094tetr001 (KUM); 19.II.2013: cvb097tetr004 (KUM); 21.II.2013: cvb106st001 (KUM), cvb106st002 (KUM), cvb106st003 (CSMNH), cvb106st004 (CSMNH); 03.III.2013: cvb178tetr003, cvb178tetr005 (both FMNH); 20.III.2014: cvb594tetr002 (KUM); 31.III.2014: cvb676tetr009 (KUM); 06.IV.2013: cvb537tetr001, cvb537tetr004 (both KUM); 07.IV.2014: cvb701tetr015 (KUM); 18.IV.2014: cvb730tetr010, cvb730tetr011 (KUM); 22.IV.2014: cvb734tetr005 (KUM). A detailed list of specimens together with collection and depository information is given in S1 Table.

**Diagnosis:** Reddish brown but head darker; eyes extremely large; antennae long, as long as head, pronotum and elytra combined; all segments longer than wide; elytral surface weakly granulate-punctate. Aedeagus and paramere are illustrated in Fig 3.

**Distribution:** Mexico, Panama (type locality), Costa Rica.

**Host records:** 122 of the genetically analyzed specimens stem from host emigrations, eight from host raids and one from an *E*. *dulcium crassinode* refuse site (S1 Table). Previously described host records of *T*. *laticeps* include *E*. *vagans*, *E*. *mexicanum* and *E*. *dulcium* [19]. Our host records confirm a preference for these *Eciton* species (Fig 4A): *T*. *laticeps* was found in 10 out of 11 *E*. *dulcium crassinode* colonies (48 individuals), 7 out of 11 *E*. *mexicanum* s. str. colonies (41 individuals), and 6 out of 8 *E*. *vagans angustatum* colonies (21 individuals). *Tetradonia laticeps* was the predominant species found in these host species (Fig 4B). We rarely found individuals associated with other host ants: 2 out of 13 *E*. *burchellii foreli* colonies (3 individuals), 3 out of 12 colonies of *E*. *hamatum* (9 individuals), 1 out of 3 *E*. *lucanoides conquistador* colony (2 individuals), and one *Neivamyrmex gibbatus* colony (4 individuals). Although this species was very abundant at the study site, it co-occurred infrequently with other *Tetradonia* species (Fig 4C). A detailed list of host records can be found in S1 Table.

#### *Tetradonia* cf. *marginalis* Reichensperger, 1935, comb. rev.

*Tetradonia marginalis* Reichensperger, 1935: 215 (original description) [40]; Jacobson & Kistner, 1998: 176 (redescription) [19].

*Tetradonia prosequens* Reichensperger, 1935: 215 (original description) [40].

*Orphnebius marginalis*: Pace, 2008: 335 (change of generic assignment) [41]; Hlaváč et al., 2011: 62 (catalogue) [21].

**Specimens examined:** 17 specimens were morphologically examined. All specimens were collected at LSBS on various collection dates: 08.II.2013: cvb007tetr002 (KUM); 09.II.2013: cvb015tetr001 (KUM); 12.II.2013: cvb038tetr011 (KUM), cvb038tetr015 (CSMNH), cvb038tetr016 (KUM); 14.II.2013: cvb048tetr010 (KUM); 19.II.2013: cvb097tetr001, cvb097tetr010, cvb097tetr011 (all KUM); 21.II.2013: cvb104tetr009 (FMNH); 08.III.2013: cvb213tetr001 (KUM); 19.III.2013: cvb382tetr006 (KUM); 21.III.2013: cvb399tetr006 (FMNH); 23.III.2013: cvb410tetr001 (CvB), cvb423tetr001 (KUM); 25.III.2013: cvb433tetr011 (KUM); 29.III.2013: cvb451tetr008 (KUM). A detailed list of specimens together with collection and depository information is given in S1 Table.

**Diagnosis:** Reddish brown but head darker; eyes moderate in size; antennae long, slightly longer than head, pronotum and elytra combined; all segments longer than wide; elytral surface weakly granulate-punctate. Aedeagus and paramere are illustrated in Fig 3.

**Distribution:** Mexico, Guatemala, Costa Rica, Panama, Trinidad, Ecuador, Peru, Brazil.

**Remarks:** We refer to the specimens examined in this study as *T*. cf. *marginalis*, because the redescription by Jacobson & Kistner, 1998 does not completely fit with the present material. While the description fits well for most body parts, one obvious difference is found in the shape of the paramere. Compared to *T*. *marginalis* described by Jacobson & Kistner, 1998, the apical lobe of the specimens examined here is narrower and more strongly curved, the sclerite on the apex of the velum is smaller, and the dorsal area of the median lobe has thin, large lateral projections, which are not shown in Jacobson & Kistner, 1998. Due to these discrepancies we identified the material from La Selva Biological Station as *T*. cf. *marginalis*. *Tetradonia marginalis* has been recorded widely from Mexico to Brazil, the widest distributional range in *Tetradonia* [19]. Considering the more or less narrow distributional ranges of the other congeners, "*T*. *marginalis*" (sensu [19]) could well constitute a species complex. A re-examination of *T*. *marginalis* specimens from different localities covering the distributional range is necessary to resolve the possible existence of cryptic species.

**Host records:** 114 of the genetically analyzed *T*. cf. *marginalis* specimens were collected in host emigrations, 22 specimens in raids and two from refuse deposits (S1 Table). *Tetradonia marginalis* has previously been collected from the following host species: *E*. *hamatum*, *E*. *burchellii*, *E*. *vagans*, *E*. *mexicanum*, *E*. *lucanoides*, *E*. *rapax*, *Nomamyrmex esenbeckii* [19]. Our community study indicates a clear host preference in *T*. cf. *marginalis* for *E*. *burchellii foreli* (82 individuals from 13 out of 13 colonies) and *E*. *hamatum* (34 individuals from 11 out of 12 colonies), and likely also for *E*. *lucanoides conquistador* (20 individuals from 3 out of 3 colonies; Fig 4). *Tetradonia* cf. *marginalis* showed a high prevalence in these hosts (Fig 4A), while we collected only two individuals from a single *E*. *vagans angustatum* colony emigration. No specimens were found with *E*. *dulcium crassinode* and *E*. *mexicanum* s. str. (Fig 4A). *Tetradonia* cf. *marginalis* co-occurred frequently in host colonies with *T*. *tikalensis*, *T*. *laselvensis* and *T*. *lizonae* (Fig 4C). A detailed list of host records can be found in S1 Table.

### Key to the *Tetradonia* species associated with *Eciton* army ants at La Selva Biological Station (adapted from Jacobson and Kistner, 1998)

The most reliable identification of *Eciton*-associated *Tetradonia* specimens at La Selva Biological Station is via the examination of aedeagi (Fig 3). Here we additionally present an easily usable key to identify the *Eciton*-associated *Tetradonia* species based on their external morphology. Note that the DNA barcode reference library can also be used for the same purpose.

**Table:** 

1. Elytral surface smooth, no distinct punctation	*T*. *tikalensis*
Elytral surface more or less granulate-punctate	2
2. Eyes extremely large, occupying entire sides of head	3
Eyes moderate in size, not occupying entire sides of head; head with temples	4
3. Tergite VII with raised area in males; pronotum wider (pronotal width/length ratio: 1.40)	*T*. *laticeps*
Tergite VII without raised area in males; pronotum narrower (pronotal width/length ratio: 1.26) area in males; pronotum narrower (pronotal width/length ratio: 1.26)	*T*. *lizonae*
4. Antennae shorter than head, pronotum and elytra combined; elytra weakly granulate-punctate	*T*. cf. *marginalis*
Antennae longer than head, pronotum and elytra combined; elytra strongly granulate-punctate	*T*. *laselvensis*

## Discussion

Mostly owing to the work of Carl Rettenmeyer, the communities of myrmecophiles associated with *Eciton* army ants have become known as extremely species-rich animal assemblages [9,11,13–16]. Despite the extensive collection and identification efforts dedicated to *Eciton* symbionts, it is clear that a large proportion of species still await scientific discovery [11]. The present survey uncovered two new species and several new host records for *Tetradonia* beetles, highlighting the need for more accurate methods to assess species diversity and host preferences in army ant myrmecophile communities.

The exploration of army ant myrmecophiles has been hampered by their immense biodiversity, which has been difficult to process in a timely manner with conventional taxonomic approaches [11]. To overcome this bottleneck and to streamline the process of species discovery and identification, we adopted a rapid DNA barcoding approach to sort the collected material into genetic clusters that were then inspected more carefully by taxonomic experts (see also [12]). Four complementary criteria were used to assess species boundaries in *Tetradonia* beetles: (a) genetic clustering using tree-based methods; (b) DNA barcode gaps; (c) character-based analysis of DNA barcoding data; (d) morphological inspection of specimens. Each analysis independently revealed the presence of five *Tetradonia* species associated with *Eciton* army ants at LSBS. Applying such an integrative taxonomic approach helps to avoid mistakes in species delimitation (e.g., [33,44,45]). For instance, focusing on the single mitochondrial locus *COI* alone to assess species boundaries can be misleading [33,44–46] and, similarly, relying on morphological studies alone can be problematic due to the presence of morphologically cryptic species [47–50]. In the present study DNA barcoding shows that, unlike in *Eciton*-associated *Vatesus* beetles [12], no cryptic diversity is present in *Tetradonia* beetles.

Besides diversity assessment, our community-based sampling protocol allowed us to reliably evaluate host preferences and co-occurrence patterns in *Eciton*-associated *Tetradonia* beetles. Host records of *Tetradonia* beetles have previously been compiled by Jacobson & Kistner [19], and their study reflects our findings to a large extent. Accordingly, we found a pattern of host specificity at LSBS that is similar to what has been described previously across collection sites [19]. Host specificity ranged from a strong preference for a single host species in *T*. *lizonae*, to low host specificity in *T*. *laticeps* (Fig 4A). However, for some species the host records described in the present study deviate somewhat from those described earlier by Jacobson and Kistner (1998). *Tetradonia laticeps*, for example, has previously been described to be associated with *E*. *vagans*, *E*. *dulcium*, and *E*. *mexicanum*, which indeed were the preferred hosts in our community survey. However, we additionally collected *T*. *laticeps* from colonies of *E*. *burchellii foreli*, *E*. *hamatum*, *E*. *lucanoides conquistador* and even in a colony emigration of the army ant *Neivamyrmex gibbatus*. The latter host record demonstrates that our *Eciton*-focused community survey is somewhat restricted, and ideally, future studies should include all local army ant species in a given community to fully assess host specificity of symbionts. Nonetheless, we are confident that our *Eciton*-focused sampling approach unveils host spectra appropriately for most associated symbionts, because the majority of myrmecophiles in the Neotropics have been described to interact only with host species of a single army ant genus [8,51].

In addition to a detailed assessment of host preferences, our community-based sampling approach allowed us for the first time to study *Tetradonia* co-occurrence at the colony level. For instance, *T*. *laticeps* infrequently co-occurred with other *Tetradonia* species, while *T*. cf. *marginalis* was regularly collected together with *T*. *tikalensis*, *T*. *laselvensis* and *T*. *lizonae*. The proximate mechanisms underlying these varying tendencies of different *Tetradonia* species to co-occur in a given colony remain unknown. In general, co-occurrence patterns can be governed by various factors including interspecific competition [52], environmental filtering due to abiotic factors [53], host-encounter- and compatibility-filtering in the case of symbioses [18,54], as well as stochastic processes [55,56]. Studying potential proximate mechanisms in the future, such as the host-searching behavior of different myrmecophiles (encounter filtering; e.g. [57]) or differential host aggression toward different myrmecophile species (compatibility filtering; e.g. [58]) might help unveil some of the determinants responsible for community structuring in army ant-symbiont communities.

In general, the degree of host specificity in symbionts is indicative of their level of specialization [18]. This is because specialization is considered to be a trade-off between increasing fitness of a symbiont in association with the primary host(s), and lowering fitness in association with other host(s) [18,59–65]. In this context, the behavior during host colony emigrations of the most host-specific *Tetradonia* species, *T*. *lizonae*, is particularly interesting. It was the only *Tetradonia* species that ran in the center of emigration columns among ant workers, a behavior that is associated with high levels of social integration in army ant myrmecophiles [14]. Such ‘specialized species’ (sensu [14]) generally live within the ants’ nest and do not evoke much aggression from the host ants [14]. Their high level of specialization possibly limits their ability to survive without host ants, so that some species die quickly under laboratory conditions [14]. Typical examples of specialized species are found in *Eciton*-associated rove beetles of the genera *Ecitomorpha* and *Ecitophya*. Besides the described behavioral adaptations, these staphylinids resemble their host ants in body shape and possibly also in coloration, a mimetic resemblance that might be targeted toward visual predators [66]. Each species is associated with a single host species only [66], which might reflect the trade-off between high levels of specialization and a narrow host range. In contrast, *Tetradonia* beetles are thought to lack such apparently derived morphological and behavioral adaptations and rather resemble the typical aleocharine beetle type [19]. Accordingly, they have been considered to be ‘generalized species’ (sensu [14]), i.e. species that are primarily found at the margin and end of emigration columns, that show a low level of social integration, that are readily aggressed by host ants, and that survive under broader ecological conditions [14]. It remains to be investigated in greater detail whether *T*. *lizonae* represents an exception among the ‘generalized’ *Tetradonia* beetles by sharing further characteristic behaviors described for ‘specialized species’ (sensu [14]).

By assessing army ant myrmecophile diversity and by providing tools for easy and reliable species identification, we hope to set the baseline for studies targeting the ecological and evolutionary dynamics in these species-rich host-symbiont microcosms. For example, this system offers the opportunity to study the trade-off between specialization and host niche breadth in a comparative framework by relating the level of social integration and symbiont specialization to the level of host specificity for the entire host-symbiont community.

## Supporting Information

S1 Fig Phylogeny of *Eciton*-associated *Tetradonia* beetles at La Selva Biological Station, Costa Rica. (PDF)

S2 Fig Co-occurrence of *Tetradonia* species at La Selva Biological Station, Costa Rica. (PDF)

S1 Table Specimen information. (XLS)

S2 Table PCR primers used in this study. (PDF)
