# Correction: Community Sampling and Integrative Taxonomy Reveal New Species and Host Specificity in the Army Ant-Associated Beetle Genus *Tetradonia* (Coleoptera, Staphylinidae, Aleocharinae)

**DOI:** 10.1371/journal.pone.0168957

**Published:** 2016-12-29

**Authors:** The PLOS ONE Staff

The following information is missing from the Funding section: Daniel J. C. Kronauer (DJCK) was supported by The Carl & Marian Rettenmeyer Ant-Guest Endowment Award.

In the Author Contributions section, Daniel J. C. Kronauer (DJCK) should be listed as one of the persons who wrote the paper.

Table 2 is incorrectly formatted. The authors have provided the new table below as a replacement for Table 2 in the published article. The publisher apologizes for the error.

**Table 2:** Key to the *Tetradonia* species associated with *Eciton* army ants at La Selva Biological Station (adapted from Jacobson and Kistner, 1998).

1. Elytral surface smooth, no distinct punction	*T*. *tikalensis*
Elytral surface more or less granulate-punctate	2
2. Eyes extremely large, occupying entire sides of head	3
Eyes moderate in size, not occupying entire sides of head; head with temples	4
3. Tergite VII with raised area in males; pronotum wider (pronotal width/length ratio: 1.40)	*T*. *laticeps*
Tergite VII without raised area in males; pronotum narrower (pronotal width/length ratio: 1.26)	*T*. *lizonae*
4. Antennae shorter than head, pronotum nad elytra combined; elytra weakly granulate-punctate	*T*. *cf*. *marginalis*
Antennae longer than head, pronotum and elytra combined; elytra strongly granulate-punctate	*T*. *laselvensis*
