# Correction: Community Sampling and Integrative Taxonomy Reveal New Species and Host Specificity in the Army Ant-Associated Beetle Genus *Tetradonia* (Coleoptera, Staphylinidae, Aleocharinae)

**DOI:** 10.1371/journal.pone.0213042

**Published:** 2019-02-22

**Authors:** Christoph von Beeren, Munetoshi Maruyama, Daniel J. C. Kronauer

In the Results section, there are errors in the “Description” paragraph of the subsection labelled *Tetradonia laselvensis*. The description of the antennae length is incorrect. The correct antennal description is: antennae long, longer than head, pronotum and elytra combined.

In the Results section, there are errors in the first sentence in the “Diagnosis” paragraph of the subsection labelled *Tetradonia* cf. *marginalis*. The description of the antennae length is incorrect. The correct sentence is: Reddish brown but head darker; eyes moderate in size; antennae short, slightly shorter than head, pronotum and elytra combined; all segments longer than wide but IX and X almost as long as wide; elytral surface weakly granulate-punctate.
