# Supplementary material for: Community Sampling and Integrative Taxonomy Reveal New Species and Host Specificity in the Army Ant-Associated Beetle Genus Tetradonia (Coleoptera, Staphylinidae, Aleocharinae)
Source: PLoS One. 2016 Nov 9;11(11):e0165056. doi: 10.1371/journal.pone.0165056 (PMC5102370; doi:10.1371/journal.pone.0165056)
Supplement: S1 Fig — (PDF) [file pone.0165056.s001.pdf]

S1 Figure. Phylogeny of *Eciton*-associated *Tetradonia* beetles at La Selva Biological Station, Costa Rica. A 'Randomized Axelerated Maximum Likelihood' (RAxML) analysis based on concatenated *COI*, *wg*, and *CS1AD* sequences was used for inferring the phylogenetic relationship of *Tetradonia* beetles. The tree was created using the software Geneious R9 (version 9.1.5) with the plugin RAxML (version 7.2.8). GTR GAMMA was chosen as the best-scoring nucleotide model as assessed by MEGA6 [1]. *Ecitomorpha arachnoides* (Staphylinidae: Aleocharinae: Ecitocharini), collected in a raid of *Eciton burchellii foreli*, served as outgroup. Bootstrap support values (1,000 replicates) are given at major nodes. The scale bar represents expected substitutions per site as inferred by the RAxML algorithm.

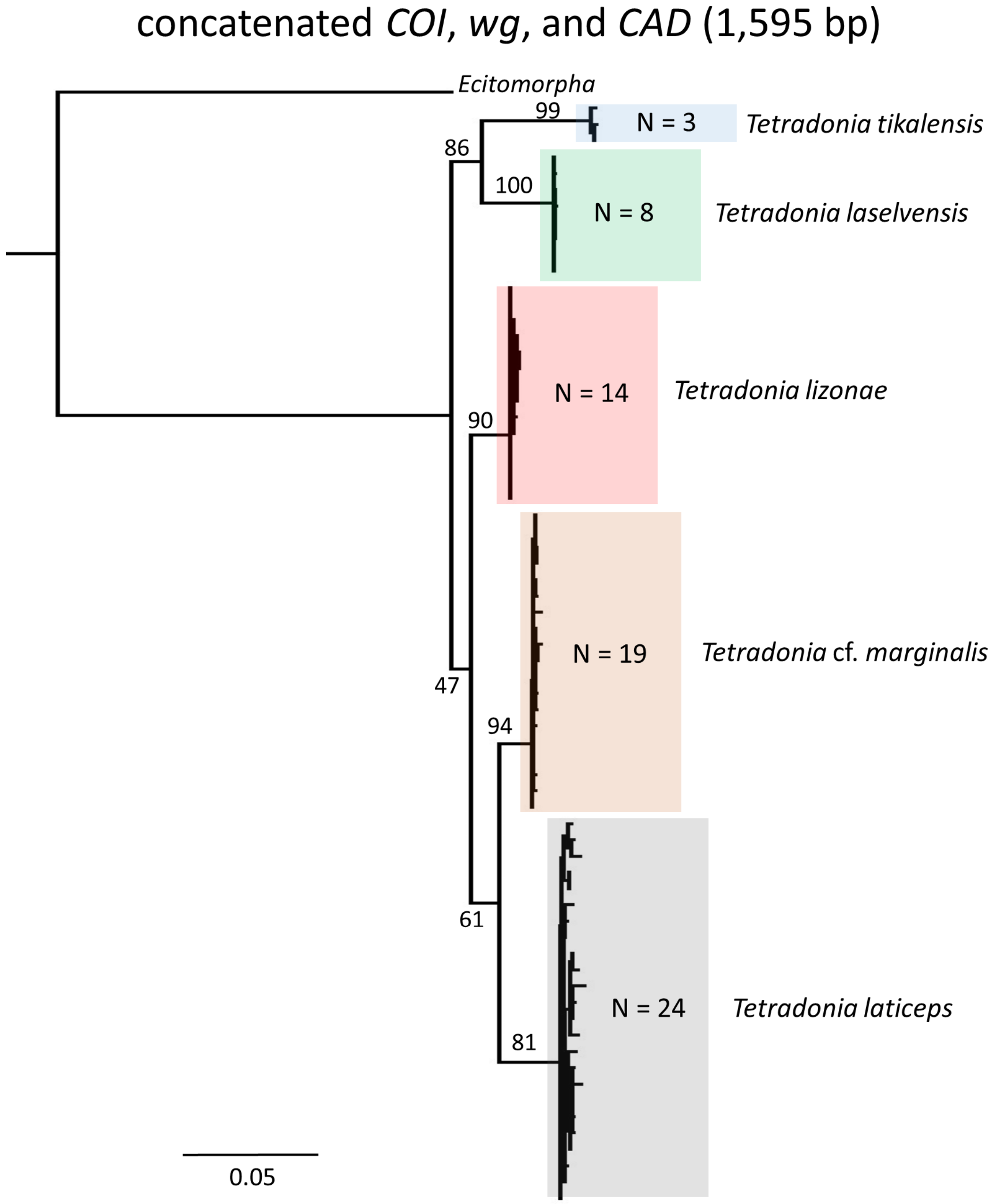

Reference

1. Tamura K, Stecher G, Peterson D, Filipski A, Kumar S. MEGA6: Molecular Evolutionary Genetics Analysis version 6.0. Mol. Biol. Evol. 2013;30:2725–9.
