# Supplementary material for: Community Sampling and Integrative Taxonomy Reveal New Species and Host Specificity in the Army Ant-Associated Beetle Genus Tetradonia (Coleoptera, Staphylinidae, Aleocharinae)
Source: PLoS One. 2016 Nov 9;11(11):e0165056. doi: 10.1371/journal.pone.0165056 (PMC5102370; doi:10.1371/journal.pone.0165056)

S2 Figure. Absolute number of *Tetradonia* co-occurrence events at La Selva Biological Station, Costa Rica. For each given species pair, white numbers depict the number of host colonies in which specimens of both species were found. For instance, *T. laticeps* and *T. marginalis* were found together in eight host colonies. Differential shading corresponds to the white numbers in cells. The white box depicts a value of zero. Abbreviations: *T. lat.* = *Tetradonia laticeps*, *T. mar.* = *Tetradonia* cf. *marginalis*, *T. tik.* = *Tetradonia tikalensis*, *T. las.* = *Tetradonia laselvensis*, *T. liz.* = *Tetradonia lizonae*.

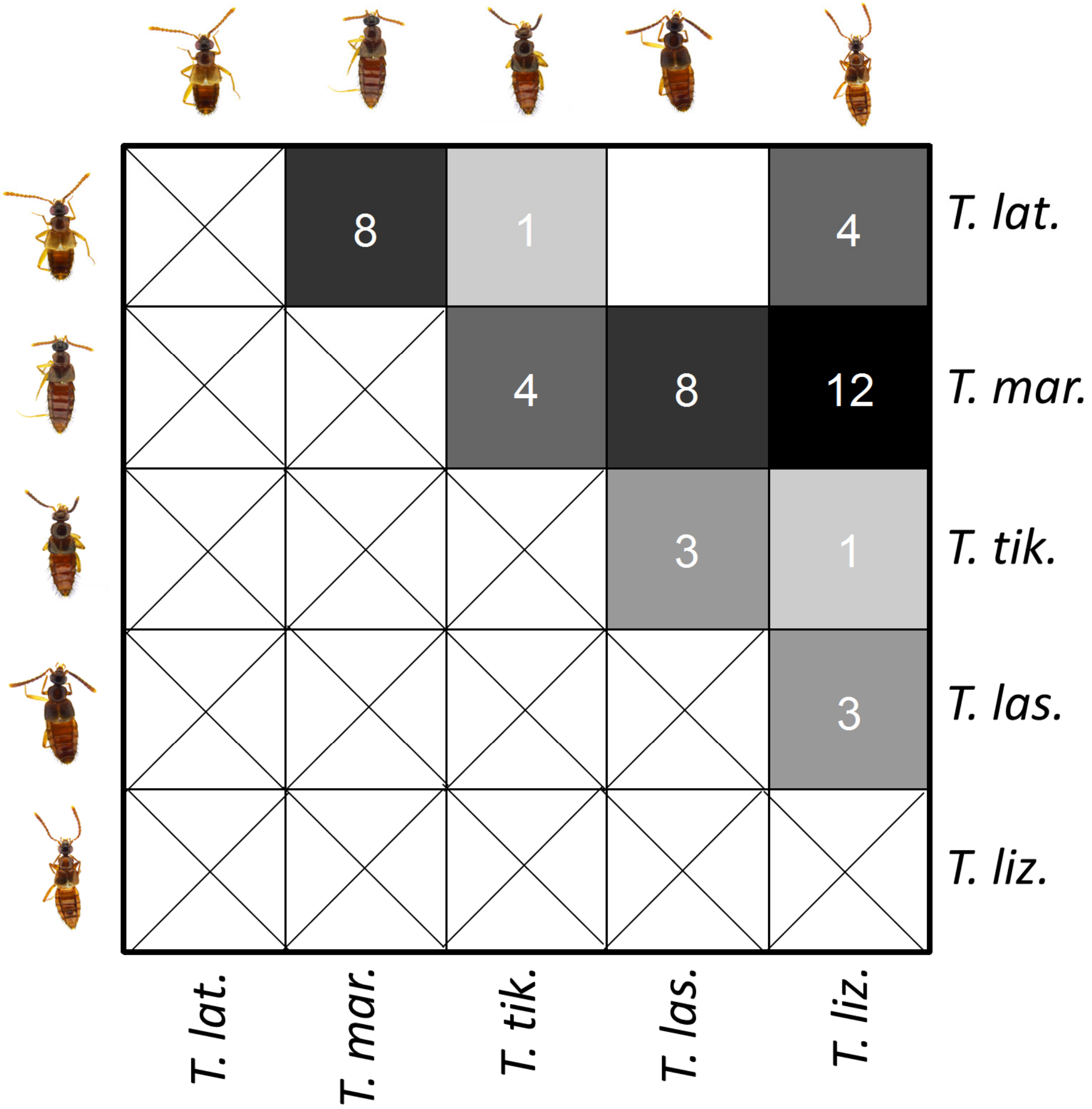

Supplement: S2 Fig — (PDF) [file pone.0165056.s002.pdf]
