# Supplementary material for: Community Sampling and Integrative Taxonomy Reveal New Species and Host Specificity in the Army Ant-Associated Beetle Genus Tetradonia (Coleoptera, Staphylinidae, Aleocharinae)
Source: PLoS One. 2016 Nov 9;11(11):e0165056. doi: 10.1371/journal.pone.0165056 (PMC5102370; doi:10.1371/journal.pone.0165056)
Supplement: S2 Table — (PDF) [file pone.0165056.s004.pdf]

S2 Table. PCR primers used in this study. Primer names in bold letters represent those primers that worked most consistently across samples for the different loci. Abbreviations: Ann. Temp. = Annealing Temperature.

Primer name	Target gene	Reading direction	Sequence (5' – 3')	Ann. Temp. range	Source
**CD667F**	*CAD*	forward	GGATGGAAGGAAGTDGARTAYGARGT	50-57°C	[1]
CD828R	*CAD*	forward	GCCATTACYTCNCCNACACTYTTCAT	50-57°C	[1]
**CD843R_Tetra**	*CAD*	reverse	TTTGAAGAGGCTTTTCARAAAGC	50-57°C	This study
CD851R	*CAD*	reverse	GGATCGAAGCCATTHACATTYTCRTCHACCAT	50-57°C	[1]
**LCO1490**	*COI*	forward	GGTCAACAAATCATAAAGATATTGG	48-55°C	[2]
**HCO2198**	*COI*	reverse	TAAACTTCAGGGTGACCAAAAAATCA	48-55°C	[2]
Wg550F	*wingless*	forward	ATGCGTCAGGARTGYAARTGYCAYGGYATGTC	50-60°C	[1]
Wg578F	*wingless*	forward	TGCACNGTGAARACYTGCTGGATG	50-60°C	[1]
**Wg578F_Tetra**	*wingless*	forward	TGCACGGTGAAGACSTGCTGGATG	50-60°C	This study
**WgAbrZ**	*wingless*	reverse	CACTTNACYTCRCARCACCARTG	50-60°C C	[1]

References

1. Wild AL, Maddison DR. Evaluating nuclear protein-coding genes for phylogenetic utility in beetles. Mol. Phylogenet. Evol. 2008;48:877–91.

2. Folmer O, Black M, Hoeh W, Lutz R, Vrijenhoek R. DNA primers for amplification of mitochondrial cytochrome c oxidase subunit I from diverse metazoan invertebrates. Mol. Mar. Biol. Biotechnol. 1994;3:294–9.
